# Complete chloroplast genome of *Dunaliella salina* CS-265 from Lake Suzie, Northern Territory, Australia

**DOI:** 10.1128/mra.00237-26

**Published:** 2026-07-17

**Authors:** Asura Khanam Lisa, Wayne G. Reeve, Damian Laird, Abha Chopra, Navid Reza Moheimani

**Affiliations:** 1 School of Environmental and Conservation Sciences, Murdoch University, Perth, Western Australia, Australia; 2 Department of Biotechnology and Genetic Engineering, Gopalganj Science and Technology University 421965, Gopalganj, Bangladesh; 3 School of Molecular and Life Sciences, Curtin University 1649, Perth, Western Australia, Australia; 4 School of Mathematics, Statistics, Chemistry, and Physics, Murdoch University, Perth, Western Australia, Australia; 5 IIID, Medical Genomics Core, Precision Medicine Centre, Murdoch University, Perth, Western Australia, Australia; University of Maryland School of Medicine, Baltimore, Maryland, USA

**Keywords:** *Dunaliella salina*, chloroplast genome, plastome, genome assembly

## Abstract

We report the complete chloroplast genome of *Dunaliella salina* strain CS-265, isolated from the hypersaline waters of Lake Suzie, Central Australia. The circular plastome is 294,897 bp, has 31.8% GC content, and encodes 101 genes (66 protein-coding, 29 transfer RNA, and 6 ribosomal RNA). This genome expands plastid resources for inland hypersaline isolates.

## ANNOUNCEMENT

*Dunaliella salina* is a halotolerant green microalga notable for its ability to accumulate high levels of β-carotene under hypersaline and high-light conditions, making it an important model for carotenoid biology and biotechnology (1). While several *D. salina* chloroplast genomes have been described, plastid genomic resources from inland hypersaline lakes of Central Australia remain limited. Here, we report the complete chloroplast genome of *D. salina* strain CS-265, originally isolated from Lake Suzie, Northern Territory, Australia.

Strain CS-265, originally isolated from Lake Suzie, Northern Territory, Australia, in 1992, and maintained by the Australian National Algae Culture Collection (ANACC, CSIRO, Australia), was obtained for this study. Cultures were grown in 3.5% NaCl supplemented f/2 medium at 25°C (pH 7.5) under a 12:12-h light:dark photoperiod (60 µmol photons m⁻² s⁻¹). Single colonies were established on 1% f/2 agar under 120 µmol photons m⁻² s⁻¹ and transferred to liquid medium for biomass propagation prior to DNA extraction. Genomic DNA was isolated using a 2% CTAB-based method (2).

Whole-genome sequencing was performed using Oxford Nanopore Technologies on a PromethION 2 platform with an R10.4.1 (FLO-PRO114M) flow cell. Libraries were prepared using the Oxford Nanopore Technologies (ONT) ligation native barcoding genomic DNA kit (SQK-NBD114.24) without DNA shearing or additional size selection. Raw signal data were basecalled using Dorado v0.7.4.14. Read quality assessment using NanoPlot generated 264,403 reads with a read N50 of 9,148 bp (3).

**Fig 1:**
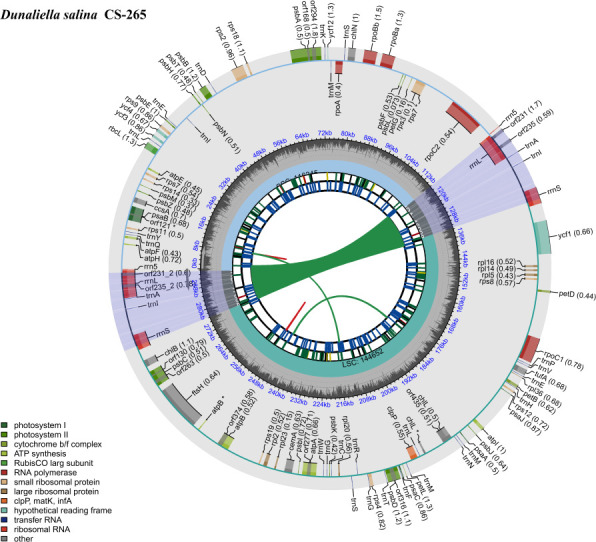
Circular map of the *Dunaliella salina* CS-265 chloroplast genome. The genome map was generated using CPGView (7). Genes on the forward and reverse strands are displayed on the outer and inner tracks, respectively, and are color-coded by functional category. The large single-copy (LSC), small single-copy (SSC), and inverted repeat (IR) regions are indicated. The gray histogram represents GC content variation, and the innermost red and green curve represents GC skew across the genome.

To enrich chloroplast-derived sequences, ONT reads were mapped against the published *D. salina* strain SQ chloroplast genome (KX530454) using minimap2 v2.24 (4). Of the 264,403 reads generated, 4,888 mapped to the reference plastome and were extracted for downstream assembly. Duplicate reads were removed using Dedupe v38.84, and error correction and read normalization were performed using BBNorm v38.84 prior to assembly (5). The filtered read set was assembled *de novo* using Flye v2.9.1, which automatically generated a single circular chloroplast contig without manual overlap trimming (Fig. 1). The final chloroplast genome assembly had an average coverage depth of 83.81×. The genome start position was subsequently adjusted during annotation based on published *Dunaliella salina* chloroplast genomes (6).

Assembly quality was evaluated using QUAST v5.3.0 (8), which confirmed a single-contig assembly with 97.58% genome fraction relative to the reference plastome (GenBank KX530454). The complete chloroplast genome of *D. salina* CS-265 is 294,897 bp in length with a GC content of 31.8%. Genome annotation was performed by annotation transfer in Geneious Prime 2025.1 using published *Dunaliella salina* chloroplast genomes as references, validated using GeSeq and manual BLAST curation. The annotated genome contains 101 genes, including 66 protein-coding genes, 29 transfer RNA genes, and 6 ribosomal RNA genes. Structural features typical of *D. salina* plastomes were observed, including a trans-spliced *psaA* gene.

## Data Availability

The complete chloroplast genome sequence of *Dunaliella salina* strain CS-265 has been deposited in GenBank under accession number PV943366, associated with BioSample SAMN49745163. The raw Oxford Nanopore sequencing reads have been deposited in the National Center for Biotechnology Information Sequence Read Archive under accession number SRR35566088, associated with BioSample SAMN51783953. All records are linked under BioProject PRJNA1099393.
